# Cardiosphere-derived cells do not improve cardiac function in rats with cardiac failure

**DOI:** 10.1186/s13287-017-0481-x

**Published:** 2017-02-15

**Authors:** Taís Hanae Kasai-Brunswick, Andréa Rodrigues da Costa, Raiana Andrade Quintanilha Barbosa, Bruna Farjun, Fernanda Cristina Paccola Mesquita, Danúbia Silva dos Santos, Isalira Peroba Ramos, Grazielle Suhett, Guilherme Visconde Brasil, Sandro Torrentes da Cunha, José Oscar R. Brito, Juliana do Amaral Passipieri, Adriana Bastos Carvalho, Antonio Carlos Campos de Carvalho

**Affiliations:** 1 Institute of Biophysics Carlos Chagas Filho, Federal University of Rio de Janeiro, Av. Carlos Chagas Filho, n°373, room G2-053, CEP:21941-902 Rio de Janeiro, RJ Brazil; 2 National Institute of Cardiology, Rua das Laranjeiras, n°374—Laranjeiras, CEP:22240-006 Rio de Janeiro, RJ Brazil; 3 National Center for Structural Biology and Bioimaging—CENABIO, Federal University of Rio de Janeiro, Av. Carlos Chagas Filho, n°373, buiding M, CEP:21941-902 Rio de Janeiro, RJ Brazil; 4 National Institute of Science and Technology for Regenerative Medicine, Av. Carlos Chagas Filho, n°373, CEP:21941-902 Rio de Janeiro, RJ Brazil

**Keywords:** Cell therapy, Myocardial infarction, Heart failure, Stem cells, Cardiosphere-derived cells, Bioluminescence

## Abstract

**Background:**

Heart failure represents an important public health issue due to its high costs and growing incidence worldwide. Evidence showing the regenerative potential of postmitotic heart tissue has suggested the existence of endogenous cardiac stem cells in adult hearts. Cardiosphere-derived cells (CDC) constitute a candidate pool of such cardiac stem cells. Previous studies using acute myocardial infarction (MI) models in rodents demonstrated an improvement in cardiac function after cell therapy with CDC. We evaluated the therapeutic potential of CDC 60 days after MI in a rat model.

**Methods:**

CDC were obtained from human discarded myocardial tissue and rat hearts by enzymatic digestion with collagenase II. At 10–15 days after isolation, small, round, phase-bright cells (PBCs) appeared on top of the adherent fibroblast-like cells. The PBCs were collected and placed on a nonadherent plate for 2 days, where they formed cardiospheres which were then transferred to adherent plates, giving rise to CDC. These CDC were characterized by flow cytometry. Wistar rats were submitted to MI through permanent occlusion of the anterior descending coronary artery. After 60 days, they were immunosuppressed with cyclosporine A during 10 days. On the third day, infarcted animals were treated with 5 × 10^5^ human CDC (hCDC) or placebo through intramyocardial injection guided by echocardiogram. Another group of animals was treated with rat CDC (rCDC) without immunosuppression. hCDC and rCDC were stably transduced with a viral construct expressing luciferase under control of a constitutive promoter. CDC were then used in a bioluminescence assay. Functional parameters were evaluated by echocardiogram 90 and 120 days after MI and by Langendorff at 120 days.

**Results:**

CDC had a predominantly mesenchymal phenotype. Cell tracking by bioluminescence demonstrated over 85% decrease in signal at 5–7 days after cell therapy. Cardiac function evaluation by echocardiography showed no differences in ejection fraction, end-diastolic volume, or end-systolic volume between groups receiving human cells, rat cells, or placebo. Hemodynamic analyses and infarct area quantification confirmed that there was no improvement in cardiac remodeling after cell therapy with CDC.

**Conclusion:**

Our study challenges the effectiveness of CDC in post-ischemic heart failure.

## Background

Heart failure (HF) represents an important public health issue due to its high costs and growing incidence worldwide. An ageing population and the increased survival rate of patients suffering coronary events contribute to aggravate this scenario [1]. Pharmacological therapy only attenuates disease progression, and patients in advanced stages of HF require heart transplantation, a treatment option that is compromised by the shortage of organs.

Recent evidence showing the regenerative potential of postmitotic heart tissue suggested the existence of a pool of endogenous cardiac stem cells in adult hearts [2]. Since then, the scientific community has dedicated enormous effort to the identification and isolation of these cells [3–5], which may ultimately provide an alternative therapy in the treatment of cardiac disease.

Cardiosphere-derived cells (CDC) constitute a candidate pool of such cardiac stem cells. CDC comprise a heterogeneous population of cardiac cells, including a potentially clonogenic stem cell subpopulation [6]. Several studies have proposed that CDC have cardiomyogenic differentiation potential in vitro [6–9]. Furthermore, infarcted animals treated with CDC showed improvement in cardiac function, reduced scar size, and increased viability of the myocardium when compared with placebo-treated animals [6, 7, 10–12].

However, work utilizing lineage-tracing has demonstrated that CDC failed to differentiate into cardiomyocytes in vivo [13] and that cell therapy did not improve cardiac function in murine infarct models [13, 14]. In addition, other authors have questioned the differentiation potential of CDC in vitro [15]. Despite existing controversies about the therapeutic applicability of CDC in animal models, these cells have already entered a phase I clinical trial (CADUCEUS) [16]. Results from the trial, after a 1-year follow-up, described a subtle enhancement in the viability of cardiac muscle without benefit to cardiac function [17]. A meta-analysis of several cardiac clinical trials, including CADUCEUS, showed that cell therapy provided no benefit in terms of left ventricular function [18].

In light of this controversy, we evaluated the therapeutic potential of cell treatment with CDC in a rat model of HF. We demonstrate that treatment with rat CDC (rCDC) or human CDC (hCDC) did not improve cardiac function in rats, and we propose that further studies utilizing animal models are needed before this new therapeutic alternative can be efficaciously applied to humans.

## Methods

### Human samples

Human myocardial tissue was obtained from the National Institute of Cardiology (Brazil). Right atrial appendages, routinely discarded from patients undergoing coronary artery bypass grafting (CABG), were collected and used for cell isolation.

### Animals

Two-month-old Wistar rats weighing ~200 g were used in this study. The animals were maintained in cages with food and water ad libitum and 12 h/12 h light/dark cycle.

### Cell isolation

Briefly, human discarded myocardial tissue or rat hearts were fractionated into small pieces (1 mm^3^) and digested using collagenase type II (200 IU/ml). Samples were submitted to 5–7 cycles of digestion for 5 min at 37 °C under gentle agitation. Cells were plated using the following culture medium: Ham’s F12 supplemented with 10% fetal bovine serum, 1% penicillin/streptomycin, 10 ng/ml of basic fibroblast growth factor (bFGF), 0.005 U/ml of erythropoietin, and 0.2 mM of glutathione. Leukemia inhibitory factor (LIF) (10 ng/ml) was added in the case of rat CDC. After ~15 days, phase-bright cells (PBCs) were collected using phosphate buffer saline (PBS) at 4 °C. PBCs were cultured using the same medium on nonadherent plates, resulting in the formation of cardiospheres. After 2 days, cardiospheres were collected and placed on adherent plates, giving rise to CDC. Cells were expanded for at least three passages before the experiments.

### Flow cytometry

CDC were dissociated using a nonenzymatic solution and blocked in PBS with 0.5% bovine serum albumin (BSA) and Fc receptor blocking reagent (BD Biosciences) for 20 min at 4 °C. Cells were then stained, using the same solution, with the following antibodies: hCDC—CD105, CD90, CD73, CD44, CD166, CD54, CD146, CD45, CD19, CD14, CD34, CD31, CD33, CD133, and CD117 (all from BD Pharmingen); rCDC—CD90 (eBiosciences), CD29, CD11b (BD Pharmingen), and CD45RA (Caltag). Data were acquired in BD FACSCanto II or BD FACSAria IIu and analyzed using FlowJo version X.

### Echocardiography

Echocardiography was performed using the Vevo 770 High-Resolution Imaging System (VisualSonics) under isoflurane anesthesia. Images were acquired in bidimensional mode and analyzed by a blinded investigator. Left ventricular end-diastolic volume (EDV), end-systolic volume (ESV), ejection fraction (EF), and fractional area change (FAC) were calculated using Simpson’s method. In brief, these parameters of cardiac function were evaluated in a long parasternal axis view and four high-temporal resolution B-mode short-axis images, taken at different ventricular levels, as described previously [19]. Examinations were performed before myocardial infarction (MI), 1 day before cell injection, and 60 and 90 days after cell injection.

### Myocardial infarction and cell injection

Animals were anesthetized with 80 mg/kg of ketamine and 20 mg/kg of xylazine and placed under positive pressure ventilation. After thoracotomy, the heart was exposed and the left anterior descending (LAD) coronary artery was ligated using 6–0 Prolene suture. The chest was closed and the animals were carefully removed from ventilation support. Sham-operated rats were submitted to an identical procedure except for LAD ligation. MI was confirmed by electrocardiography (ECG). Infarcted animals were included in the study only if the EF was below 45%.

Sixty days after MI, 5 × 10^5^ CDC were delivered to the myocardium using echocardiogram-guided injection. Cells were injected using a 29 G needle into two regions of the infarct border zone (2.5 × 10^5^ cells/injection). The placebo-treated group received only vehicle, which was 25% Matrigel® (Corning) in Ham’s F12. In the case of the hCDC group and the corresponding placebo group, immunosuppression with cyclosporine A was started 2 days before cell injection at a dose of 10 mg/kg/day [20] and maintained for 10 days. In order to evaluate the quality of cells before injections, viability was analyzed using Trypan Blue Solution 0.4%. More than 95% of the cells were viable before injection.

### Bioluminescence

CDC were transduced with Luciferase 2 using a lentiviral vector as described previously [21]. To validate our injection method, hCDC and rCDC were stably transduced with luciferase 2 for in-vivo tracking. Cell tracking of CDC-Luc2 was performed after intramyocardial injection guided by echocardiogram. The groups of animals used for cardiac function evaluation received nontransduced CDC instead. In-vivo bioluminescence studies were conducted using the IVIS Lumina Imaging System (Xenogen Corporation) after intraperitoneal injection of 150 mg/kg of d-Luciferin (Caliper Life Sciences). Animals were imaged from the first day after CDC injection until the luminescent signal reached the lower limit of detection for the equipment (600 counts). Analyses were performed using Living Image software version 3.2.

### Langendorff perfusion experiments

Animals received 5 IU/g of heparin by intraperitoneal injection and were euthanized. Hearts were removed by extended sternotomy and cannulated through the ascending aorta. The mechanical function was preserved using retrograde perfusion (at 10 ml/min with a peristaltic pump) with Krebs–Henseleit solution (NaCl 118 mM, KCl 4.7 mM, NaHCO_3_ 25 mM, KH_2_PO_4_ 1.2 mM, MgSO_4_ 1.2 mM, glucose 11 mM, and CaCl_2_ 1.25 mM) in a modified Langendorff system. This physiological saline solution was maintained at 37 °C and bubbled with carbogenic gas mixture (95% O_2_, 5% CO_2_) for oxygenation and maintenance of pH at 7.4. A small latex balloon attached to a pressure transducer (MLT0380; ADInstruments) was inserted into the left ventricle. The balloon volume was increased until 0 mmHg of initial diastolic pressure was obtained and then 20 μL of solution was added every 2 min. The systolic and diastolic pressure records were digitized through an analog–digital interface (PowerLab 400; ADInstruments) for offline analysis with Chart 4.0 software (ADInstruments). Pressure versus volume curves were obtained at varying loads in systole and diastole, and data were analyzed by calculating the integral of the curves using MATLAB software version 7.0.

### Histomorphometry

Myocardial tissue was fixed in 4% paraformaldehyde for 24 h and paraffin-embedded. Six-micrometer sections were stained with Sirius red and scanned to obtain digital images of the entire heart. The percentage of left ventricular area occupied by the infarct was calculated by dividing the area stained in red (collagen fibers) by the total area of the left ventricle. These areas were quantified using Image Pro Plus software version 7.0.1 in three regions of the heart (apex, middle portion, and base). Results are shown as an average of the three values.

### Statistics

Data are shown as mean ± standard deviation. Echocardiography data were analyzed using two-way ANOVA, while Langendorff and histomorphometric data were analyzed using one-way ANOVA. Bonferroni’s post test was applied in both cases and *p* < 0.05 was considered significant. Graph Pad Prism software version 6.0 was used for all analyses.

## Results

### Isolation and characterization of human and rat CDC

**Fig. 1:**
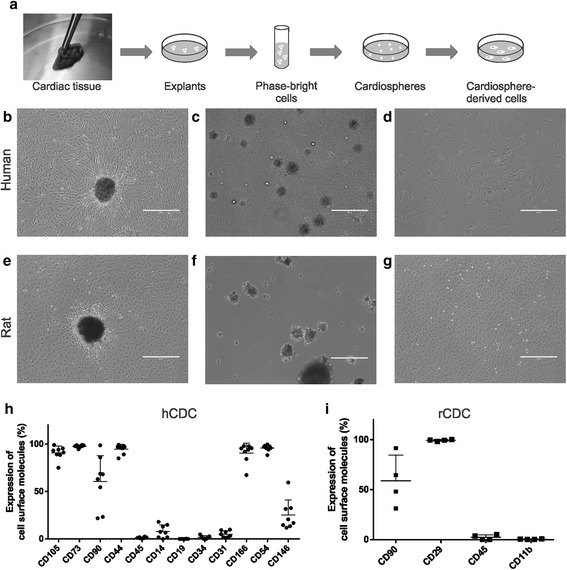
Isolation and characterization of CDC. **a** Cardiac tissue was minced into small fragments and plated as explants. PBCs were collected and cultured on nonadherent plates, forming cardiospheres. CDC were generated by transferring cardiospheres to adherent plates. Representative images of human and rat explants (**b**, **e**), cardiospheres (**c**, **f**) and CDC (**d**, **g**). Surface molecule expression was characterized by flow cytometry in hCDC (**h**) and rCDC (**i**). Note the large standard deviations for CD90 in both hCDC and rCDC, representing heterogeneous expression of this molecule. *hCDC* human cardiosphere-derived cell, *rCDC* rat cardiosphere-derived cell

We obtained CDC from human discarded myocardial tissue and rat hearts. Between 10 and 15 days after cell isolation from human and rat tissues, small, round, PBCs appeared on top of adherent fibroblast-like cells derived from explants. PBCs were collected and cultured for 2 days on a nonadherent plate, where they formed cardiospheres which were then transferred to adherent plates, giving rise to CDC (Fig. 1a). We obtained cultures of explants derived from human (Fig. 1b) and rat (Fig. 1e) tissues. Fig. 1c and f show representative images of cardiospheres derived from human and rat hearts, respectively. Both hCDC (Fig. 1d) and rCDC (Fig. 1g) were successfully isolated and expanded until the third passage. hCDC expressed mesenchymal markers (CD105, CD73, CD90) and adhesion molecules (CD54, CD146, CD166) and showed low expression of hematopoietic and endothelial markers (CD45, CD14, CD19, CD34, CD31) when analyzed by flow cytometry (Fig. 1h; *n* = 8). Similarly, rCDC expressed high levels of mesenchymal markers (CD90.1, CD29), whereas contamination by hematopoietic cells (CD45, CD11b) was minimal (Fig. 1i; *n* = 4).

### Transduction and tracking of CDC in infarcted myocardium

**Fig. 2:**
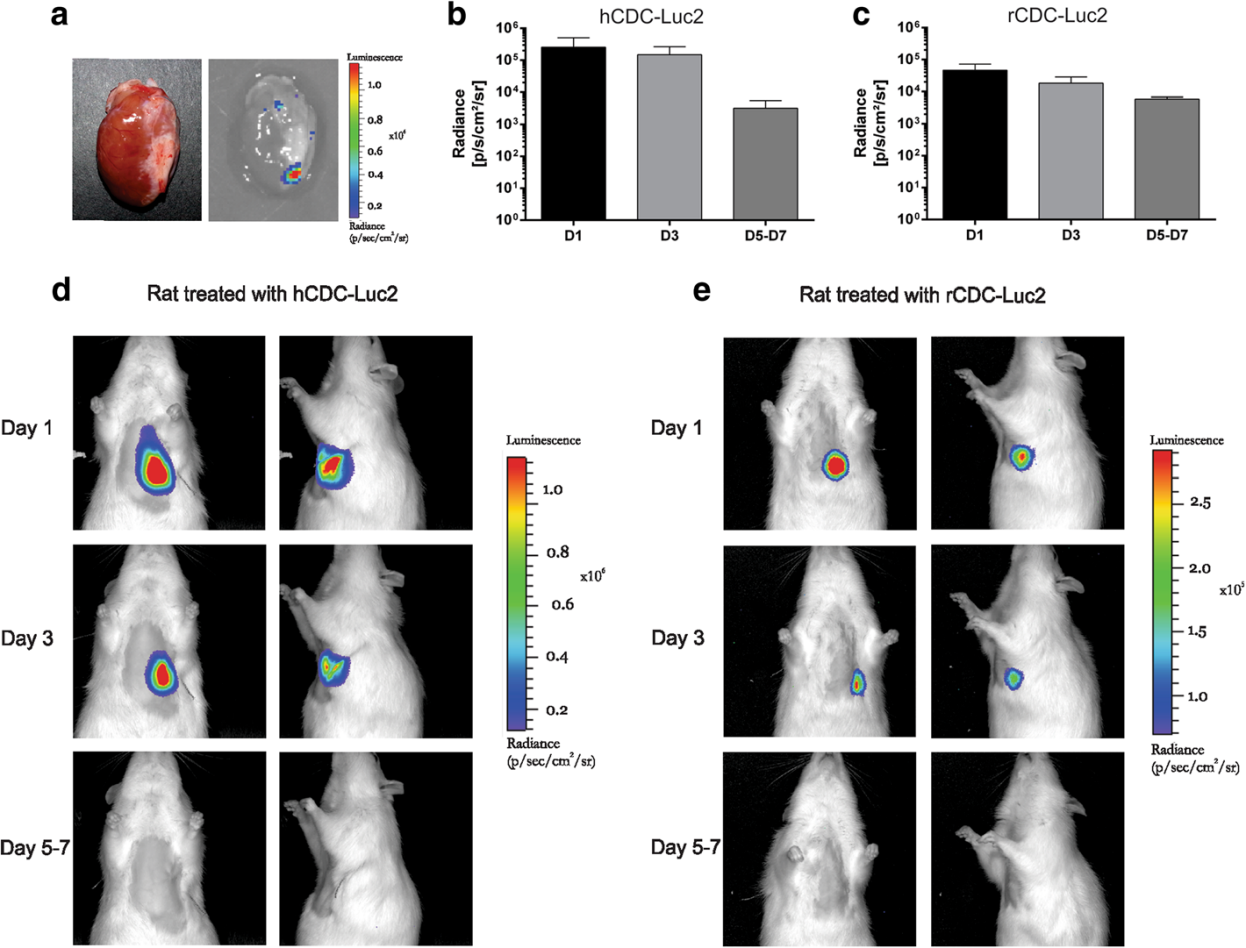
CDC tracking by bioluminescence. **a** Ex-vivo image of a rat heart after echocardiography-guided intramyocardial injection of transduced CDC. Signal is located on the border zone of the infarct. Quantification of luminescent signal in rats injected with hCDC (**b**) or rCDC (**c**). **d**, **e** In-vivo cell tracking shows luminescent signal on the precordial region of infarcted rat. Luminescence was detected until 5–7 days after injection. Color scales are shown in units of radiance, and quantification data are plotted on a log scale (Color figure online). *hCDC* human cardiosphere-derived cell, *rCDC* rat cardiosphere-derived cell

Previous work has identified CDC as endogenous cardiac progenitor cells with regenerative potential in models of acute MI [6, 9, 22, 23]. We investigated this potential in a rat chronic model by the injection of CDC 60 days post MI. Figure 2a shows a rat infarcted heart 3 days after cell therapy. Cells were present at the injection site. The bioluminescent signal of CDC-Luc2 was detected in the thoracic region until day 7 post injection (Fig. 2d, e). Radiance quantification demonstrated a 98.8% decrease in mean bioluminescent signal at 5–7 days after injection of hCDC in immunosuppressed rats (D1: 2.54 ± 2.52 × 10^5^, D3: 1.49 ± 1.15 × 10^5^, D5–7: 3.21 ± 2.34 × 10^3^p/sec/cm^2^/sr) (Fig. 2b). Syngeneic rCDC presented an 87.2% reduction in signal over the same period (D1: 4.63 ± 2.59 × 10^4^, D3: 1.82 ± 1.04 × 10^4^, D5–7: 5.95 ± 0.87 × 10^3^p/sec/cm^2^/sr) (Fig. 2c).

### Cardiac functional evaluation post CDC treatment

**Fig. 3:**
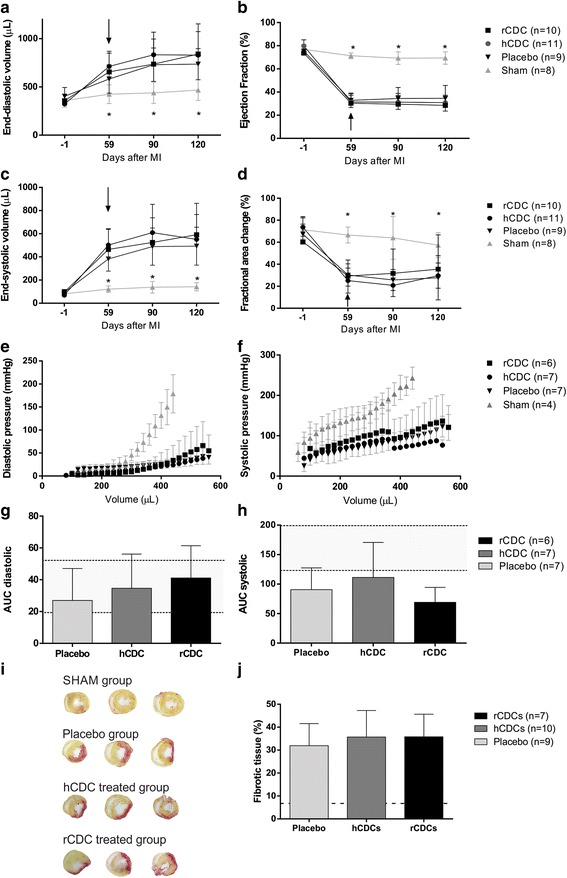
Cardiac function evaluation after CDC therapy. **a** EDV, (**b**) EF, (**c**) ESV, and (**d**) FAC. *Arrows*, rats treated 1 day after the data were collected. No differences were found when placebo, rCDC, and hCDC groups were compared. Sham-operated rats had significantly lower values of EDV and ESV and higher values of EF and FAC when compared with the infarcted groups. Considering equal volumes, infarcted groups had lower values of diastolic (**e**) and systolic (**f**) pressures when compared with sham-operated rats, suggesting cavity dilation and systolic dysfunction respectively. The area under the diastolic (**g**) and systolic (**h**) curves (*AUC*) was calculated. There were no differences between placebo and cell-treated groups. **i** Representative images of serial heart tissue slices stained by Sirius Red from sham, infarcted animals treated with CDC and placebo. Fibrotic tissue is marked in *red*. **j** Quantification of fibrotic area showed a significant increase in infarcted groups when compared with sham-operated rats. *Shaded area* represents values belonging to the sham group. No differences were observed between placebo, rCDC, and hCDC groups. *p < 0.05 when comparing sham animals with infarcted animals (Color figure online). *hCDC* human cardiosphere-derived cell, *rCDC* rat cardiosphere-derived cell

To evaluate potential benefits of hCDC and rCDC therapy, we performed in-vivo and ex-vivo analyses of cardiac function. Figure 3 and Table 1 show the results of echocardiographic examinations. We observed no differences in EDV (Fig. 3a), EF (Fig. 3b), ESV (Fig. 3c), or FAC (Fig. 3d) between hCDC, rCDC, and placebo-treated animals at all time points assessed. In these animals, all tested parameters of cardiac function were significantly lower than those observed in sham-operated animals.

**Table 1:** Cardiac function of experimental groups

		Experimental group			
Parameter	Time (days)	Sham (*n* = 8)	Placebo (*n* = 9)	hCDC (*n* = 11)	rCDC (*n* = 10)
Ejection fraction (%)	–1	76.85 ± 4.24	75.50 ± 1.48	79.83 ± 5.34	72.97 ± 3.69
	59	70.72 ± 2.08	32.88 ± 6.28*	31.48 ± 7.26*	30.39 ± 6.97*
	90	69.33 ± 5.45	34.49 ± 9.48*	31.26 ± 7.66*	29.56 ± 7.59*
	120	69.44 ± 5.36	34.62 ± 11.08*	30.89 ± 6.39*	28.58 ± 7.68*
End-diastolic volume (μl)	–1	358.8 ± 68.61	383.7 ± 80.47	323.2 ± 39.71	357.7 ± 65.29
	59	424.4 ± 97.15	643.1 ± 98.04*	712.3 ± 137.9*	656.8 ± 213.1*
	90	438.7 ± 110.40	730.6 ± 172.90*	833.3 ± 234.3*	736.3 ± 259.8*
	120	467.7 ± 108.2	736.20 ± 161.6*	830 ± 243.9*	840.2 ± 313.4*
End-systolic volume (μl)	–1	80.91 ± 22.78	93.20 ± 25.63	69.52 ± 16.13	96.06 ± 18.68
	59	121.4 ± 28.45	382.4 ± 105.2*	502 ± 135.8*	464.40 ± 179.3*
	90	137.5 ± 50.67	488.80 ± 160.5*	609.2 ± 244.8*	525 ± 212.6*
	120	142 ± 34.97	492.5 ± 163.6*	551 ± 214.9*	591.40 ± 271.3*
Fractional area change (%)	–1	71.38 ± 11.53	67.38 ± 9.06	73.75 ± 8.34	66.05 ± 8.87
	59	66.58 ± 7.23	30.22 ± 10.12*	25.15 ± 11.40*	24.90 ± 10.79*
	90	63.95 ± 19.47	25.70 ± 9.56*	20.78 ± 10.16*	25.85 ± 8.36*
	120	57.33 ± 11.30	27.71 ± 20.11*	29.65 ± 11.46*	25.47 ± 9.69*

Data presented as mean ± SD. Day –1 shows baseline values (before myocardial infarction) for all groups. Day 59 shows data 1 day before treatment with placebo, hCDC, or rCDC

*p < 0.05 compared with sham-operated group

*hCDC* human cardiosphere-derived cell, *rCDC* rat cardiosphere-derived cell

To investigate the hemodynamic performance of isolated hearts after cell therapy with CDC, we used a modified Langendorff apparatus. The diastolic pressure versus volume curves of infarcted animals shifted downwards when compared with the sham-operated group (Fig. 3e). The same was observed for the systolic curve (Fig. 3f). The area under the curves was analyzed and no differences were observed between placebo and cell-treated animals (Fig. 3g, h).

To assess whether CDC treatment reduced the infarcted area, we performed morphometry with Sirius red staining. Figure 3i shows the sham-operated heart with viable muscle (light staining), whereas the infarcted heart shows collagen fibers (dark staining), indicating scar areas. The percentage of fibrosis was quantified in the left ventricle after CDC cell therapy. Treated animals (hCDC or rCDC) had no improvement in the amount of viable cardiac muscle when compared with the placebo group (Fig. 3j). The sham-operated group had significantly less fibrotic tissue than the other groups.

## Discussion

The present study evaluated the efficacy of late CDC treatment in a rat model of HF. We chose to base our work on a late treatment because it more closely resembles the situation of most HF patients who develop the disease over the course of many years. Moreover, in advanced stages there is no definitive therapy other than a heart transplant. To date, the potential benefits of CDC therapy had not been evaluated in a rat chronic model. We reproduced in detail all of the steps described previously for stem cell isolation, identification, and injection. Despite these efforts, we show that CDC therapy did not improve cardiac function or reduce scarring in this animal model.

We isolated, expanded, and characterized CDC derived from discarded human tissue or rat hearts using established procedures [6, 9, 15]. Cells had the immunophenotypic profile described previously [6, 9] except for low c-kit expression. The low expression of c-kit observed in our work should not have posed a problem, because recent work has shown no correlation between the presence of c-kit and the regenerative efficacy of CDC [24]. Much like the CDC used in previous studies [16, 17], our cells expressed heterogeneous levels of CD90.

We injected CDC into the peri-infarcted area of the myocardium, because this was the most common delivery route in rodent acute MI models treated with CDC [6, 22, 24, 25]. Several authors have reported that a majority of injected cells die a few days after the procedure, and that 10% or fewer of the cells remain in the heart [11]. Despite the chosen cell dose (2 million cells/kg of body weight) being higher than the dose administered in the CADUCEUS clinical trial (178,000–357,000 cells/kg of body weight), our treatment resulted in low cell engraftment and retention levels, comparable with those described previously [14]. To exclude the possibility that the expression of luciferase 2 could alter the quality and a putative therapeutic effect of CDC, the tracking assays with transduced CDC were performed in a separate group of animals. One limitation of our study was the delivery of cells in only two injection sites. Although injected cells are known to migrate from the site of injection, we cannot exclude that multiple sites of injection are necessary to improve cardiac function.

Several groups have reported improvements in cardiac function after CDC therapy in rodent models [6, 9, 22–26]. Acute treatment of small animals with CDC reduces scar size [22, 25, 26], increases EF [6, 9, 23, 24, 26] and muscle viability [9, 23], and decreases EDV as well as ESV [25]. On the other hand, chronic treatment performed in large animal models yielded subtle or negative results [10–12]. In 2009, Marban’s group showed that placebo-treated and CDC-treated pigs using the intracoronary route had similar values of EF [10]. In a later study, the same group reported preservation of function, but not improvement, when administering intramyocardial injection of CDC [11]. Takehara et al. [12] achieved improved ventricular function in post-MI pigs using a combination of bFGF gels and CDC, an approach less translatable to the clinical setting; however, administration of CDC to pigs without additional growth factors resulted in only slight improvement to the EF.

In preclinical experiments performed in small animals, the best results have been obtained when cell therapy has been administered immediately after coronary occlusion, whereas the benefit obtained when using chronic models in large animals has been quite modest. Despite this limited success achieved in animal model studies, CDC were administered to humans 2–4 months after MI in the CADUCEUS phase I clinical trial. One year after treatment, the trial reported MRI results showing a decrease in scar tissue. Nevertheless, a recent meta-analysis of several clinical trials reported that the CDC used in CADUCEUS did not yield benefits in terms of clinical events or changes in cardiac function [18]. Similarly, all other cell therapies evaluated to date have been of limited benefit to MI patients. The disappointing results from human trials clearly do not reflect the improvements observed in acute animal models.

Despite our efforts to closely reproduce best-practice conditions for CDC therapy, evaluation of the animals showed no differences in cardiac function between groups receiving human cells, rat cells, or a placebo. This lack of difference was also evident with regards to scar size. Previous work in mice has similarly failed to demonstrate any improvement after CDC treatment in an acute MI model, in spite of achieving long-term engraftment. In that study, functional evaluations were performed by several methods, such as echocardiogram, hemodynamic analysis, MRI, and positron emission tomography [14]. In addition, other groups have cast doubt on whether CDC indeed constitute a source of stem cells with cardiomyogenic potential [13, 15].

In light of these discrepant findings in animal models, we suggest that it is time for a cautious step back. Pending issues—such as the differentiation potential of CDC, the existence of CDC subpopulations, and new approaches to the isolation of cardiac stem cells [27]—must be addressed before the potential therapeutic effect of CDC in heart disease can be realized.

## Conclusion

Treatment with CDC did not improve heart function or prevent heart chamber dilatation in rats with MI and cardiac remodeling. Our study challenges the effectiveness of CDC in post-ischemic HF.

## Abbreviations

bFGF: Basic fibroblast growth factor
BSA: Bovine serum albumin
CABG: Coronary artery bypass
CADUCEUS: CArdiosphere-Derived aUtologous StemCElls to reverse ventricUlar dySfunction
CDC: Cardiosphere-derived cells
ECG: Electrocardiography
EDV: End-diastolic volume
EF: Ejection fraction
ESV: End-systolic volume
FAC: Fractional area change
hCDC: Human cardiosphere-derived cells
HF: Heart failure
LAD: Left anterior descending
LIF: Leukemia inhibitory factor
MI: Myocardial infarction
PBC: Phase-bright cell
PBS: Phosphate buffer saline
rCDC: Rat human cardiosphere-derived cells
